# The effect of targeted exercise on knee-muscle function in patients with persistent hamstring deficiency following ACL reconstruction – study protocol for a randomized controlled trial

**DOI:** 10.1186/s13063-018-2448-3

**Published:** 2018-01-26

**Authors:** Bo Bregenhof, Uffe Jørgensen, Per Aagaard, Nis Nissen, Mark W. Creaby, Jonas Bloch Thorlund, Carsten Jensen, Trine Torfing, Anders Holsgaard-Larsen

**Affiliations:** 1 Orthopaedic Research Unit, Department of Orthopaedics and Traumatology, Odense University Hospital, Institute of Clinical Research, University of Southern Denmark, Sdr. Boulevard 29, 5000 Odense C, Denmark; 2 Department of Sports Science and Clinical Biomechanics, University of Southern Denmark, Campusvej 55, 5230 Odense M, Denmark; 3 Department of Orthopaedics, Lillebaelt Hospital, Kolding, Skovvangen 2-8, 6000 Kolding, Denmark; 4 School of Exercise Science, Australian Catholic University, PO Box 456, Virginia, Queensland 4014 Australia; 5 Department of Radiology, Odense University Hospital, Sdr. Boulevard 29, 5000 Odense C, Denmark

**Keywords:** ACL reconstruction, Rehabilitation, Muscle strength, Physical function, Exercise

## Abstract

**Background:**

Anterior cruciate ligament (ACL) reconstruction, using hamstring auto-graft is a common surgical procedure, which often leads to persistent hamstring muscle-strength deficiency and reduced function. The purpose of this randomized controlled trial (RCT) is to investigate the effect of a combined, progressive, strength and neuromuscular exercise intervention on knee muscle strength, functional capacity and hamstring muscle-tendon morphology in ACL-reconstructed patients with persistent hamstring muscle-strength deficiency compared with controls.

**Methods/design:**

The study is designed as a multicenter, parallel-group RCT with balanced randomization (1:1) and blinded outcome assessments (level of evidence: II) and will be reported in accordance with the CONSORT Statement. Fifty ACL-reconstructed patients (hamstring auto-graft) with persistent limb-to-limb knee-flexor muscle-strength asymmetry at 12–24 months’ post surgery, will be recruited through outpatient clinics and advertisements. Patients will be randomized to a 12-week progressive, strength and neuromuscular exercise group (SNG) with supervised training twice weekly or a control intervention (CON) consisting of a home-based, low-intensity exercise program. Outcome measures include between-group change in maximal isometric knee-flexor strength (primary outcome) and knee-extensor muscle strength, hamstring-to-quadriceps strength ratios of the leg that has been operated on and Knee injury and Osteoarthritis Outcome Score (KOOS) (secondary outcomes).

In addition, several explorative outcomes will be investigated: The International Knee Documentation Committee Subjective Knee Form (IKDC), the Tegner Activity Score, rate of force development (RFD) for the knee flexors and extensors, tendon regeneration and potential muscle hypertrophy at graft harvest site evaluated by magnetic resonance imaging (MRI), postural control, kinetic/kinematic gait characteristics and knee-related functional capacity.

**Discussion:**

This RCT is designed to investigate the effect of combined, progressive-resistance and neuromuscular exercises on knee-flexor/extensor strength, in the late rehabilitation phase following ACL reconstruction. Reduced hamstring strength represents a potential risk factor for secondary ACL rupture and accelerated progression of osteoarthritis. If deemed effective, the intervention paradigm introduced in this study may help to improve current treatment strategies in ACL-reconstructed patients.

**Trial registration:**

ClinicalTrials.gov, ID: NCT02939677 (recruiting). Registered on 20 October 2016.

**Electronic supplementary material:**

The online version of this article (10.1186/s13063-018-2448-3) contains supplementary material, which is available to authorized users.

## Background

Anterior cruciate ligament (ACL) reconstruction is a common arthroscopic procedure, with approximately 300,000 reconstructions performed annually in the United States [1]. ACL reconstruction aims to restore functional stability of the knee, and can be performed using a variety of different surgical techniques and graft sites [2]. The hamstring tendon is one of the most commonly used graft donor sites used for ACL reconstruction [1, 3]. Although current ACL reconstruction procedures intend to restore internal knee biomechanics, function of the ACL-reconstructed knee remains different from that of healthy knees [4, 5] and is associated with early development of osteoarthritis [6–8]. Therefore, information about factors associated with increased risk of osteoarthritis, such as lower-limb muscle-strength deficits, should be part of the risk management with ACL reconstruction [6]. In a recent study, maximal isometric hamstring-muscle strength was reported to be 22% lower in the ACL-reconstructed limb at 18 months post surgery, and reduced knee-joint function was also observed compared with healthy controls [9]. Notably, hamstring muscles are considered important protagonists to the ACL [10] and reduced knee-flexor strength represents a potential risk factor for secondary ACL rupture [11]. According to international standards and consensus, ACL postoperative rehabilitation is generally limited to the first 9–12 months post surgery. Furthermore, the effect of early (first 12 months post surgery) rehabilitation has previously been studied [12, 13] .Thus, long-term rehabilitation protocols of ACL-reconstructed patients, especially when using semitendinosus auto-grafts, are strongly advised [14]. Due to well-documented positive effects, neuromuscular training has become an integral part of most early post-operative ACL reconstruction rehabilitation programs [12, 15–17]. However, there is limited evidence on studies performing muscle-strength interventions during the late rehabilitation phase (12 months post surgery) following ACL reconstruction [12, 14].

Tissue regeneration by means of magnetic resonance imaging (MRI)-verified muscle volume and tendon-graft-site regrowth is generally considered to be one of the pivotal preconditions for postoperative recovery in terms of improved knee-joint function [18, 19]. Several studies have examined the regenerative capacity of the semitendinosus and gracilis tendons [20–22] demonstrating substantial tendon regeneration at 6 months after time of harvesting. However, regeneration is slow and may continue up to approximately 12–24 months after ACL reconstruction, without guarantee of full muscle-tendon regeneration [20, 22], and thus it may have impact on the effect of muscle-strength interventions.

The objective of this study is, therefore, to investigate the effect of targeted exercise on knee-muscle strength and joint function in ACL-reconstructed patients with persistent hamstring muscle deficiency 12–24 months post surgery compared with controls. Furthermore, an explorative part of the study will evaluate the extent of tissue regeneration at the graft harvest site by MRI, and kinematic and kinetic analyses on functional gait performances.

As such, this study is expected to provide important clinical evidence on late-phase rehabilitation in ACL-reconstructed patients. If deemed effective, these study findings may help identify and refine optimal rehabilitation paradigms for ACL-reconstructed patients and help to describe and/or develop optimal exercise-based therapy to improve donor-site tendon regeneration.

## Methods/design

### Study design

**Fig. 1:**
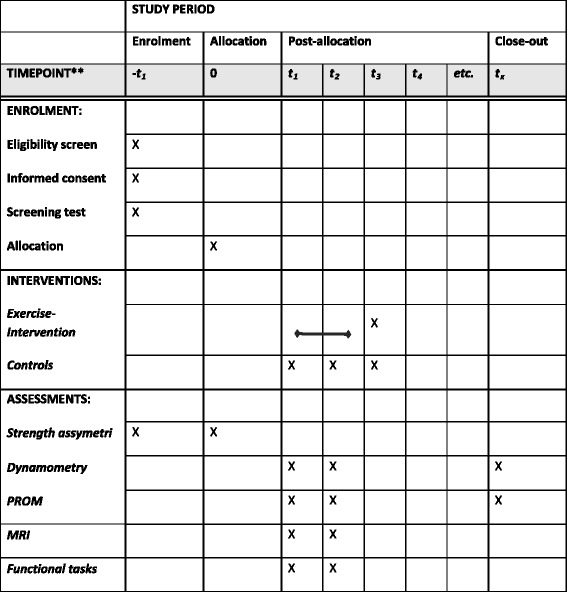
SPIRIT Figure. Template of content for the schedule of enrollment, interventions and assessments

The study is designed as a prospective, superiority, parallel-group randomized controlled trial (RCT) with balanced and blinded randomization (1:1) with blinded outcome assessment (level of evidence: II). The study protocol adheres to the SPIRIT Statement (Standard Protocol Items: Recommendations for Interventional Trials) (see Additional file 1 for the SPIRIT Checklist and Fig. 1 for the SPIRIT Figure) as well as to the CONSORT Statement (Consolidated Standards of Reporting Trials) [23, 24].

### Participants, randomization and blinding

A sample of 50 elective, ACL-reconstructed patients are planned to be recruited at 12–24 months’ post-operative outpatient clinic follow-up from the Department of Orthopaedics and Traumatology, Odense University Hospital, Denmark and the Department of Orthopaedics, Lillebaelt Hospital, Kolding, Denmark, and from poster advertisement at local sports clubs, education facilities, etc.

Eligible patients (inclusion and exclusion criteria are listed in Table 1) will receive verbal and written information about the conditions of the trial and sign a standardized consent form. The primary investigator will orally introduce the trial to eligible participants. Following this, interested patients will receive written information and an invitation to be screened using handheld dynamometry for final evaluation of eligibility. Inclusion criteria will be confirmed from the patient’s written medical history, obtained from the surgeon, as well as during conversation with the patient. Handheld dynamometry will be used to determine objective eligibility with respect to between-limb strength asymmetry. Height and weight will be measured to determine Body Mass Index (BMI). If participants meet the inclusion criteria, they will be invited to participate in the study.

**Table 1:** Inclusion and exclusion criteria for participants in the study

Inclusion	Exclusion
• ACL reconstruction using hamstring tendon auto-graft	• Other known joint pathology that will affect participation in the intervention
• Age between 18 and 40 years	• BMI > 35
• A pathologically defined between-limb asymmetry ratio (> 10% leg-to-leg difference) for maximal isometric strength of the knee flexors at 12–24 months’ follow-up	• Decline to participate • Not understanding written Danish language • Other known medical conditions that will affect participation in the intervention

*ACL* anterior cruciate ligament, *BMI* Body Mass Index

Written informed consent will be given prior to, or at, baseline testing and collected by the primary investigator or the study coordinator/study nurse. Finally, participants will have the option of supplementary informed discussion with the primary investigator at any time point prior to baseline testing. Patients declining to participate in the trial will receive standard healthcare instructions (specified below).

After baseline measurements, participants will be randomly allocated (permuted blocks of four to six persons) to either the targeted exercise or the control group. The randomization sequence will be computer generated using Stata 13.0 (StataCorp, College Station, TX, USA) statistical software with a 1:1 allocation ratio using sequentially numbered opaque, sealed envelopes. The allocation sequence and preparation of the concealed envelopes will be completed by a central study coordinator (JL) not involved in the conduct of the trial. To prevent bias during the allocation sequence, the name and date of birth of the participant will be written on the envelope immediately after randomization by the research nurse. The primary investigator will be blinded to allocation and will not participate in testing, randomization or in the training of study participants. The statistical analysis will be performed on allocation codes only and thus the data analysts will be blinded in relation to intervention allocation.

Blinding to treatment allocation of patients, physiotherapists and nurses (healthcare providers) will not be possible due to the nature of the interventions. However, blinded, independent data collectors will be responsible for baseline and follow-up assessments, and responses entered in databases identified by identification numbers only. The principal investigator and data analyst (BB) will be blinded to treatment allocation as data will be analyzed using coded identification numbers. The coding and re-coding of the identification numbers will be performed by the central study coordinator.

To maintain the overall quality and legitimacy of this clinical trial, un-blinding in terms of allocation, will only occur in exceptional circumstances (e.g., harm) when knowledge of the actual treatment is essential for further management of the participant. Investigators will before un-blinding, discuss with the members of the projects advisory group whether un-blinding is necessary and, to which extent, the un-blinding unfolds. The primary investigator will maintain the blind as far as possible. Allocation will not be disclosed to other study personnel including other site personnel, monitors, corporate sponsors or project office staff. The investigator will report all code breaks (with reason) as they occur.

### Combined strength and neuromuscular exercise intervention (SNG)

Participants allocated to combined muscle strengthening and neuromuscular exercise (SNG) will be engaged in an exercise regimen based on progressive strength training, including elements of neuromuscular exercise. The training program is based on exercises described in the current academic literature which have been applied to ACL-reconstructed patients [16, 25–28] (Additional file 2). Furthermore, advice on exercises from professional experts in physiotherapy, ACL-reconstruction rehabilitation and knee-joint biomechanics, have been implemented. No isolated development or feasibility work of the present exercise program has been developed. Implementation in accordance with “best practice” has been undertaken.

The SNG intervention will be performed twice weekly for 12 weeks with each session lasting 60–70 min. Patients will be admitted continuously into class-based groups of both novice and experienced participants. Group-based exercises will have a maximum of six participants, and will be performed at the hospital rehabilitation facility (Kolding) as well as in a local commercial fitness centre (Odense). The physiotherapists involved in the training are experienced in the rehabilitation of knee-related injuries, will participate in scientific seminars on ACL-rehabilitation and exercise, and will be instructed and trained in the specific intervention protocol by the principal investigator prior to the initiation of participant recruitment.

After 2 weeks of familiarization with emphasis on correct technique, the strengthening part of the intervention will commence consisting of eight exercises (Additional file 2) for the lower extremities performed in three sets of 10 repetitions with an intensity of 12 repetitions maximum with the time for rest (between sets). To apply with the principles of explosive-type resistance training (RFD-training) the participants will be instructed to complete the concentric phase of the movement “as fast as possible,” then pause briefly, and complete the eccentric phase of the movement in approximately 2–3 s. Measurements of the velocity during the concentric phase are not applicable; however, the quality of the explosive component of the exercise is supervised by an experienced physiotherapist throughout the entire intervention period. The participants are encouraged to perform the maximum number of repetitions possible within each set. If the number of repetitions is below 8 or exceeds 12, the loading will be adjusted for the next set. The physiotherapists will supervise the individual progression for each participant.

The neuromuscular aspects of the training program will focus on proprioception and postural function with the key elements being balance and functional stability [29]. To allow for progression of the neuromuscular exercises, two or three levels of difficulty are given (Additional file 2). Progress is made when a given exercise is performed with good sensorimotor control and a high quality of performance (based upon visual inspection by the physiotherapist). Number of sets, reps and weight will be recorded to determine whether the patient is ready to progress after each session. Acceptable compliance is defined as participation in 75% or more of all training sessions (i.e., 18 sessions).

### Control group (CON)

Controls (CON) will receive instructions (a pamphlet) regarding a training regimen of home-based, weight-bearing, low-intensity exercises (Additional file 3). CON participants will be instructed and advised to perform the home-based exercises twice weekly. Specific exercise instructions using body weight (gravity) and resistance bands, will be provided by the physiotherapists upon randomization (Additional file 3). The home-based training regimen of the control group (CON) is based upon the fact that persistent asymmetry of hamstring muscle strength has been evaluated by hand-held dynamometry, before enrollment. There is currently, no established national guideline, concerning late rehabilitation programs, to ACL patients with muscle asymmetry and/or knee-instability symptoms. However, since the referring surgeon has observed pathological asymmetry of the knee-extensors at inclusion and for ethical reasons, patients in the current trial are offered a low-resistance exercise regimen to mimic realistic clinical guidelines for the current patient group (Additional file 3).

### Pain monitoring during exercise intervention

The intervention procedures may provoke musculoskeletal pain and participants will, therefore, be asked to rate perceived pain intensity in their training diary before and after training and test sessions using a Visual Analogue Scale (VAS, 0 mm = no pain, 100 mm = worst possible pain). Pain (muscle or joint) up to a level of 50 mm will be considered “acceptable” in the period immediately after each training session. The day after training, pain should subside to “pain as usual” and not increase over time. “Pain as usual” is defined as the pain level prior to exercise. If this does not occur, the level of exercise progression will be reduced [25].

Participants in the CON group will have access (phone) to the involved staff for advice throughout the duration of the trial.

All loads (kg) lifted during all exercises will be recorded in an exercise diary, comprising the date of each session (to determine the number of sessions), exercises performed (including loads lifted, number of repetitions and sets), perceived exertion (Borg RPE CR-10) [30]. Furthermore, SNG participants will be instructed to note the individual resistance and level of difficulty for the neuromuscular exercises (to determine progression). During the study, no concomitant care or interventions are prohibited.

### Timing of assessments

**Fig. 2:**
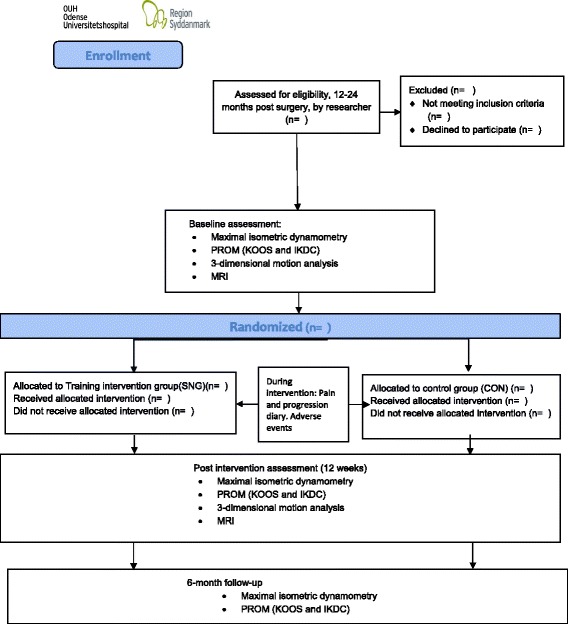
Study flowchart. Participant flow through intervention period

Assessments will be performed at baseline (prior to randomization), following the intervention (12 weeks post baseline) (the primary endpoint) and 6 months post intervention (Fig. 2; Table 2: Outcome measurements). Subjects will be evaluated in terms of full range of motion, and knee laxity, though proprioceptive status is not evaluated before inclusion. Although testing for quadriceps-muscle-strength deficit is part of the study, eligible participants having only quadriceps muscle-strength deficits will not be included. After the intervention period, all participants will be encouraged to continue the exercise program unsupervised at home or in their local fitness center.

**Table 2:** Outcome measurements

	Data collection instrument	Collection time point		
		Baseline	Post (12 weeks)Primary endpoint	Post (6 months)
Primary outcome				
Maximal isometric knee flexor strength (of the ACL-reconstructed leg)	Maximal isometric dynamometry	x	x	x
Secondary outcomes				
KOOS – 5 subscales	PROM	x	x	x
Maximal isometric knee-extensor strength	Maximal isometric dynamometry	x	x	x
Hamstring-to-quadriceps ratio	Maximal isometric dynamometry	x	x	x
Explorative outcomes				
Counter movement jump	3-dimensional motion analysis	x	x	
Gait analysis	3-dimensional motion analysis	x	x	
One-legged jump for distance	Simple functional test	x	x	
Postural sway	Simple functional test	x	x	
Rate of force development	Maximal isometric dynamometry	x	x	x
Quadriceps and hamstring morphologies Volume Peak cross-sectional area Length	MRI	x	x	
IKDC	PROM	x	x	x

Patient characteristics (age, BMI, time since operation, Tegner Activity Level Scale score) will be obtained at baseline

*KOOS* Knee injury and Osteoarthritis Outcome Score, *IKDC* International Knee Documentation Committee Subjective Knee Form, *MRI* magnetic resonance imaging, *PROM* Patient-reported Outcome Measure

### Patient characteristics

At baseline (prior to randomization), height and weight will be measured, and age will be recorded. Time since surgery will be obtained from the Danish National ACL Reconstruction Registry and the Tegner Activity Level Scale will be completed.

### Primary outcome measure

The primary outcome is the between-group change in maximal unilateral isometric knee-flexor strength (hamstring) recorded in the leg that has been operated on using stabilized dynamometry at a 90° angle (0° = full anatomical extension), according to methods described by Jensen et al. [31] and Holsgaard-Larsen et al. [9]. In general, excellent test-retest reliability in lower-limb muscle strength has been reported in both healthy people and patients [32, 33].

### Secondary outcome measures

Between-group changes in maximal unilateral isometric extensor strength (quadriceps) and hamstring-to-quadriceps-muscle-strength ratio will be obtained using the same type of stabilized dynamometry as used for the primary outcome variable [9, 32, 34, 35]. Furthermore, the Knee injury and Osteoarthritis Outcome Score (KOOS) questionnaire will be administered to all trial participants to assess their perception of daily knee function and related symptoms [36, 37]. KOOS is a 42-item, self-administered, self-explanatory questionnaire that covers five patient-relevant categories: Pain, Other Disease-Specific Symptoms, Activity of Daily Living Function, Sport and Recreation Function and Knee-related quality of life. It has been developed and validated for several cohorts of young and/or active patients with knee injury and/or knee osteoarthritis [36–39].

### Explorative outcome measures

The International Knee Documentation Committee Subjective Knee Form (IKDC) will be used. The IKDC is a site-specific instrument that has been designed to assess symptoms, function, and sports activity levels in patients who have one or more of a variety of knee conditions including ligamentous, meniscal, articular cartilage, arthritic and patello-femoral pathologies [38, 40].

The Tegner Activity Score will also be employed, as it aims to provide a standardized method of grading work ability of the lower limb, performance of activities of daily living and magnitude of competitive sport participation, in patients with orthopedic knee injuries and knee osteoarthritis. The scale score ranges from 0 (knee-related sick leave or disability) to 10 (engaged in competitive sports). The Tegner Activity-level Scale has shown acceptable test-retest reliability in knee patients [41], and been shown to be valid and reliable for assessing activity level in individuals with ACL injury [38, 41].

Rapid muscle force capacity (rate of force development: RFD_200_), representing the rate of force change during the very early phase of muscle contraction (0–200 millisecond (ms) relative to force onset), will be determined for the knee flexors and extensors [32, 42, 43].

Three-dimensional kinematic/kinetic analysis of horizontal gait at self-selected velocity and standardized one- and two-legged (dual-force-plate methodology) counter-movement jumping (CMJ) will be performed using an eight-camera motion capture system (100 Hz; Vicon Motion Systems, Oxford, UK), in synchrony with two force plates (1000 Hz; AMTI, 0R6-7 Series Inc., Watertown, MA, US) embedded in the floor. Bilateral CMJ will be performed with each leg positioned on a separate force plate, while unilateral CMJ analysis on a single force plate will be undertaken in accordance with the procedures described previously [44]. Using the standard plug-in-gait marker model and inverse dynamics analysis [44, 45], angle and moments of the lower-limb joints and be calculated [9, 46].

Postural control is evaluated by assessment of the movement of the center of pressure (CoP) of the vertical ground reaction force within the base of support of the feet to maintain postural equilibrium during the static stance. Deficits in postural sway have been reported after ACL injury and reconstruction [29]. Patients will be instructed to stand one-legged, on the test limb with the contralateral limb flexed and both arms on the hips and maintain a stable posture on the platform during which the range of CoP excursion (30 s) is recorded and subsequently analyzed [47]. The test will be performed for both legs, with eyes open and closed.

The one-legged hop for distance mimics ambulant sporting activities and demands explosive muscle function, postural balance ability, and functional stability of the knee. This test has previously been used as a sensitive and responsive measure in ACL research [9, 48, 49] and previous studies have reported high test-retest reliability in trials with patients suffering from ACL deficiency [9, 50–52].

The participant stands on the leg to be tested, then takes off to cover a maximal horizontal distance, and lands on the same limb with hands placed behind the back. The participant is carefully instructed to perform a maximal horizontal hop with a controlled and balanced landing and to keep the landing foot in place for 2 to 3 s, until the landing position has been recorded by the tester. Failure to maintain one-legged standing balance for 3 s results in a disqualified hop. The distance hopped is measured in centimeters (±0.5 cm) from the toe at push-off to the heel where the participant lands. Participants will perform one practice trial and at least three test trials or until no further improvement is observed. The best trial will be used, and a symmetry index will be calculated (reconstructed side/non-affected side).

Tendon regeneration (semitendinosus tendon) and changes in macroscopic hamstring and quadriceps muscle morphology (hypertrophy) will be assessed by MRI. Evaluation will be performed for all participants from both groups prior to, and after, the 12-week intervention period.

MRI scans will range from the iliac crest orthogonal axial plane to the transition between the proximal tibial metaphysis and diaphysis, while participants lie supine in the scanner (Philips Inginia 1.5 T system with software release R5.1.17). MRI sequence details will be the following: Coil = Integrated posterior/anterior coil; sequence, transverse T1-weighted mDIXON TSE, TR (repetition time) 550 ms; TE (echo time) 20 ms; 1 NSA (number of signals averaged); FOV (field of view) 340 × 467 mm., ST (slice thickness) 10 mm; gap 3 mm; number of slices 60. Post-processed images will be In Phase (IP) and Water Only (W). Both the radiologist and primary investigator will, prior to analysis, measure a subgroup of MRIs to achieve data on inter-observer reliability. All evaluations will be blinded to the participant’s randomization. In case of disagreement on the radiographic findings, a consensus opinion will be obtained. All MRI evaluations will be performed in collaboration with the Section of Musculoskeletal MRI, at the Department of Radiology, Odense University Hospital. Hamstring and quadriceps volume, selected single-site, axial, cross-sectional area (CSA) values, and hamstring length will be evaluated by MRI analysis as described by Eriksson et al. [20] and Tadokoro et al. [21]. The morphological characteristics (volume, peak CSA and length) of the quadriceps and hamstring muscles will be evaluated for both limbs using manual segmentation by tracing the margin of the respective muscle and tendon in successive axial slices. Furthermore, length of tendon and muscle will be determined by transversal slice. Measurements will be made of the hamstring muscles, including semitendinosus, gracilis, semimembranosus and the long head of the biceps femoris. Quadriceps muscles will include rectus femoris, vastus intermedius, lateralis and medialis. Cross-sectional area will be determined by locating the 10-mm slice with the greatest CSA and averaging this along with five additional slices immediately cranial and caudal (in total, 11 slices). Tendon regeneration will be defined as having occurred if the tendon is visible below the musculotendinous junction. The semitendinosus and gracilis tendons will be identified, and evaluated in terms of volume, peak CSA and length, from the distance between the joint line and the distal muscle-tendon junction. Tendon regeneration will be evaluated as being full, partial or non-regenerated, in comparison with the ipsilateral leg [19, 22].

### Adverse events

Adverse events will be monitored with a non-leading questionnaire during the entire phase of intervention, as a part of participant’s training diary. All events will be coded in accordance with the Medical Dictionary for Regulatory Activities, as currently required by all regulatory authorities, including the US Food and Drug Administration and the European Agency for the Evaluation of Medicinal Products. All participants will have the opportunity to contact the primary investigator (BB) and the engaged physiotherapist(s) at any time during the trial. Adverse events or harm to participants during the intervention will be reported to the primary investigator (BB) daily. There are no stopping criteria based on the collected data. We intend to report/publish, independently of the direction of the results.

### Ethical considerations

All participants will be informed about the nature, scope and risks of the study, and will be asked to give their written consent to participate. The trial has been registered with The Regional Committees on Health Research Ethics for Southern Denmark with registration ID S-20160034. The study will be performed in accordance with the ethical standards in the 1964 Declaration of Helsinki.

Participants may withdraw from the study for any reason at any time. The primary investigator may also withdraw participants from the study to protect their safety and/or if they are unwilling or unable to comply with required study procedures. Throughout the intervention and follow-up period, participants are reminded, by email, about consecutive clinical visits. All withdrawals concerning study participation, will be reported in future publications, including incomplete outcome datasets, due to incomplete follow-up, participant discontinue or deviation from intervention.

No provision of care beyond that immediately required for the proper and safe conduct of the trial, and the treatment of immediate adverse events related to trial procedures is provided. Participants’ healthcare needs that arise as a direct consequence of trial participation (e.g., intervention-related harms), will be covered and treated accordingly, by the Danish public healthcare system. No plans are made to provide or pay for ancillary care during the trial.

All tests described in the protocol have been performed previously in a similar patient group without causing any issues and/or undesired side-effects [9]. As described above, study participants will report pain on a VAS, before and after each training session. Pain up to 2 on the scale is considered “safe,” up to 5 is considered “acceptable,” while pain scores above 5 are considered “high risk.” Post-training/-testing pain is accepted as long it does not last for more than 24 h after the previous training/test session and participants judge the pain to be acceptable.

The study will adhere to Recommendations for the Conduct, Reporting, Editing and Publication of Scholarly Work in Medical Journals (the Vancouver Convention) [53]. The authors of the current protocol article will also be co-authors on publications derived from this study relative to their specific contributions. Irrespective of positive or negative results, the data will be published in international peer-reviewed journals and presented as lectures at scientific conferences, nationally and internationally, in accordance with CONSORT guidelines for the reporting of clinical trials [23, 24]. The need for a Data Monitoring Committee (DMC) was deselected due to known minimal risks of the planned intervention procedures. Consecutive modifications to trials will be evaluated and performed by the Trial Steering Committee (TSC). Protocol modifications will be reported to, and approved by, the Steering Committee while also reported to the Regional Ethical Committee. All modifications will be communicated to all study members by the primary investigator (BB) and all modifications to the test and exercise protocols will be reported at ClinicalTrials.gov. No plans are made for ancillary studies involving the collection or derivation of data for purposes that are separate from the main trial or for ancillary studies.

### Sample size calculation and statistical procedures

Sample size estimation was performed using maximal unilateral isometric knee-flexor strength of the operated leg (primary outcome) from a previously published pilot study on the present test protocol and reliability data from our laboratory [5]. The statistical model contains one baseline and one follow-up assessment.

Between-group difference in change score of 0.31 Nm bw^-1^ in knee-flexor strength in the ACL-reconstructed limb resulting in a less than 2.5% deficit of the healthy leg prior to intervention is considered of clinical relevance [5]. To achieve a statistical power of 80% (*β* = 0.80), using a SD of 0.37 Nm bw^-1^ pre and post intervention, and allowing the detection of statistically significant differences at an *α* = 0.05 level (two-tailed testing), a sample size of *n* = 23 was calculated for each group; the estimated recruitment of 50 participants (in total) allows for possible dropouts.

All study data will be obtained electronically on site by the research physiotherapist in the laboratory where the data will originate. Original study forms will be collected, stored and entered on file at the participating site by the research nurse. Participant files are stored in numerical order in a secure and accessible place. Participant files will be maintained in storage for a period of 5 years after completion of the study. The research nurse will, weekly, send email reports with information on missing data, missing forms and missing visits. A complete back up of the primary database will be performed twice a month, to an external back-up hard drive and subsequently to a secure Share-Point location administrated by the university hospital.

All outcome measures will be checked for Gaussian distribution by use of QQ-plots and parametric statistical and/or non-parametric analyses will be used when deemed appropriate. All statistical tests will use an *α*-level of 0.05 and data will be presented as means and 95% confidence interval unless otherwise stated.

Between-group mean differences in outcome measures and 95% confidence intervals will be evaluated using a general mixed linear model in which the participant’s baseline score is entered as a covariate [54]. All analyses will follow the “intention-to-treat principle” [55]. Furthermore, subsequent “per-protocol” analysis for patients demonstrating the a-priori-defined acceptable compliance to exercise will be performed. The “last-observation-carried-forward” method will be used for data imputation in cases of missing outcome measures. All statistical analyses will be blinded to the analyst (BB) and will be performed using Stata 13 software (StataCorp, College Station, TX, USA). No plans for additional analyses is made.

### Data interpretation

To minimize bias, we have a-priori decided how to interpret different result scenarios: (1) If knee-flexor strength improvement is superior (statistically significant and clinically relevant (≥0.31 Nm bw^-1^ in knee-flexor strength)) in SNG compared with CON, the combined intervention of strength and neuromuscular exercises will be considered the preferred treatment of choice; (2) If gains in knee-flexor strength are superior in CON compared with SNG, home-based exercises will be considered the preferred treatment of choice; and (3) if knee-flexor strength improvement does not differ between the two treatment groups, the intervention associated with the greatest functional improvement and pain relief, and the least adverse events, will be favored.

## Discussion

This randomized clinical trial will evaluate the effect of a targeted resistance-exercise intervention on neuromuscular knee-joint function and muscle-tendon morphology in ACL-reconstructed patients with persistent hamstring-muscle-strength deficiency. As a prospective RCT, the results of this study are expected to provide high-level evidence of the potential clinical and functional benefits of performing an exercise-based intervention in the late rehabilitation phase following ACL reconstruction, using hamstring auto-grafts. So far, no RCTs have evaluated the effect of combined, progressive-resistance training and neuromuscular exercise in the late rehabilitation phase in patients demonstrating persistent hamstring deficiency following ACL reconstruction. If deemed effective, the intervention paradigm introduced in this study may help improve current treatment strategies for patients undergoing ACL reconstruction.

### Outcome variables

Comprising the primary outcome variable, maximal hamstring-muscle strength, immediately following intervention (12 weeks) is chosen to examine if persistent hamstring-strength deficiency can be reduced by targeted exercise-based intervention. Furthermore, patient-reported perceived knee-joint function is evaluated at 6-month follow-up to evaluate the long-term effect of the intervention on knee function and knee-related quality of life.

Patient-reported outcome variables are obtained (secondary/explorative outcomes) to investigate potential effects on self-perceived function in daily living, knee pain, symptoms, sports and recreation and knee-related quality of life. A recent cross-sectional study from our laboratory demonstrated strong associations between patient-reported outcomes and the objective outcomes listed in the current trial [9]. Such potential associations, if also detected in the current prospective RCT, may provide further understanding of the underlying impairments in neuro-mechanical muscle function associated with ACL surgery.

In accordance with the International Classification of Function, Disability and Health [56] the current test battery is composed of different test types that cover different domains. Maximal isometric muscle strength is related to *body structure and function* whereas the remaining test types (one-legged jump for distance, kinematic/kinetic outcomes of gait and counter movement jumping) mainly serve to evaluate neuromuscular impairments, which are primarily related to *activity*. Impairments in *body structure and function* (i.e., Maximum Voluntary Contraction (MVC)) are linked to limitations in *activity* [56], which have been proposed to affect health-related quality of life [57]. Thus, intervention paradigms aimed at improving maximal knee-extensor and flexor strength (MVC) might be expected to improve *activity* outcomes and thereby positively affect quality of life. In support of this notion, we have previously demonstrated that hamstring and quadriceps MVC are central outcome variables to explain the inter-individual variation in KOOS profile (subjectively perceived knee-joint function) in ACL-reconstructed patients [9]. Thus, it may be reasonable to assume that the expected improvements in hamstring and/or quadriceps-muscle strength elicited by the intervention regimen will result in improvements in the remaining three test types evaluating *activity* and patient-reported outcomes. The present choice of relevant test parameters is based on previous study reports [9, 31, 48–51, 58–64] and is commonly used in the local department of orthopedics and orthopedic/biomechanical science.

### Study design

To ensure a high internal validity and to avoid subgroup analysis due to potential differences in rehabilitation protocol(s), all participants allocated to CON intervention will be advised to perform home-based exercises of low intensity (for details, see Additional file 3). Consequently, this may affect the generalizability, especially in the conventional clinical settings where late-phase (12 months post surgery) rehabilitation generally is not offered to patients, besides brief recommendations regarding engaging in training and/or referencing to web-based rehabilitation programs.

To improve external validity and generalizability, only a few exclusion criteria will be employed. An exclusion criterion of BMI above 35 will be used since obesity causes soft skin tissue artifacts that will affect the validity of 3-dimensional motion analysis. Mechanical stability in the reconstructed knee, will be evaluated by the surgeon at the standard 1-year outpatient clinic follow-up. In case of an insufficiently healed graft, poor mechanical knee-joint stability or reduced range of motion, the surgeon will evaluate the need for re-surgery. In such cases participants will be excluded due to the potential occurrence of other known joint pathologies that may affect adherence to the intervention protocol. Patients who demonstrate associated meniscal and/or cartilage procedures, which are commonly related to ACL reconstruction, will not be excluded even though their functional limitations may be slightly different from the remaining sample.

Since the trial is based upon patients volunteering for a physical intervention, the study may potentially be affected by selection bias. However, it will be possible to compare the KOOS scores of the current sample with all patients registered in the Danish National ACL Reconstruction Registry and consequently assess potential discrepancies.

Acceptable compliance with exercise will be defined as participation in 75% or more of all training sessions conducted (i.e., 18 sessions). The current study will be based upon the “intention-to-treat” analysis including all patients allocated for training irrespective of the number of training sessions. A “per-protocol” analysis will also be performed to explore whether compliance to training will have any effect on the observed results.

### Limitations

Analysis of cost-effectiveness is not planned for this intervention. Furthermore, despite the interesting perspective of qualitative analysis concerning patient experience that could have been added to the protocol, no priority on this perspective has been obtained and is thus omitted.

Due to the non-invasive/non-pharmacological intervention, no auditing is planned during the trial. Due to a relatively low sample-size of the present mechanistic trial no analysis of cost-effectiveness is planned for this study.

## Summary

This study will use a randomized controlled design to investigate the effect of a targeted exercise intervention compared with controls, on knee-joint function in patients with persistent hamstring-muscle-strength deficiency 1 year after ACL reconstruction surgery using hamstring tendon auto-graft. The trial results should help to determine whether targeted exercise interventions can increase hamstring strength, and thereby be employed in the late rehabilitation phase for ACL-reconstructed patients demonstrating persistent hamstring muscle-strength deficiency. If deemed effective, the intervention paradigm introduced in this study may help to improve current treatment strategies and their timing for patients undergoing hamstring auto-graft ACL reconstruction. Furthermore, the explorative part of the trial will provide understanding of the underlying impairment in mechanical muscle function associated with ACL reconstruction. The results will be submitted to a peer-reviewed international journal for publication irrespectively of the outcome obtained, in accordance with the CONSORT guidelines for the reporting of clinical trials.

## Additional files

Additional file 1: SPIRIT Checklist. SPIRIT 2013 Checklist: recommended items to address in a clinical trial protocol and related documents. (DOC 123 kb)

Additional file 2: Exercise protocol. Exercise protocol for supervised intervention group. (PDF 1193 kb)

Additional file 3: Exercise protocol. Exercise protocol for home-based intervention group. (PDF 286 kb)
