# Supplementary material for: The effect of targeted exercise on knee-muscle function in patients with persistent hamstring deficiency following ACL reconstruction – study protocol for a randomized controlled trial
Source: Trials. 2018 Jan 26;19:75. doi: 10.1186/s13063-018-2448-3 (PMC5787267; doi:10.1186/s13063-018-2448-3)
Supplement: Supplementary file 2 — Exercise protocol. Exercise protocol for supervised intervention group. (PDF 1193 kb) [file 13063_2018_2448_MOESM2_ESM.pdf]

Additional file 1.

Exercises with a focus primarily on progressive strength training

Leg-press

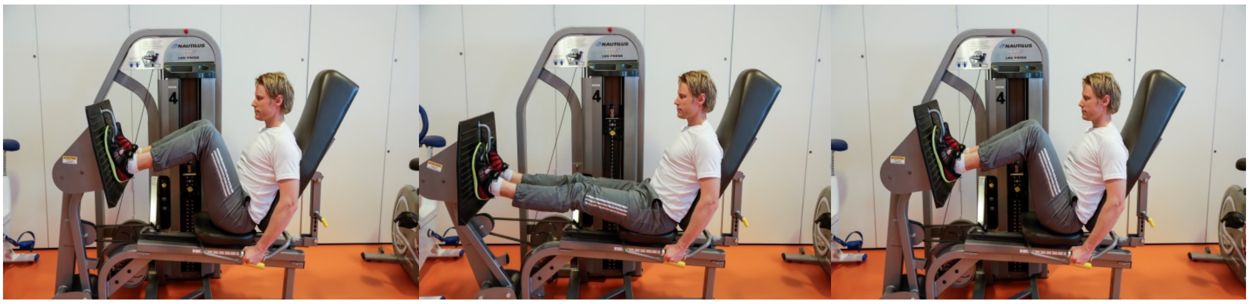

The patient is instructed to sit in the apparatus with their back against padded support; to put both legs on the platform directly in front, with their feet a shoulder width apart; to arrange the leg-press so that their torso and legs make a 90-degree angle; to press the platform all the way until their legs are fully extended in front, making sure that their knees do not lock (hyper extension); to mainly push with their heels and use their quadriceps to go back to the starting position; then, to return to the original position with flexed knees. Focus will be on a fast/forceful knee extension, followed by a slower and controlled movement towards starting position. When the patient demonstrates full control in terms of performance, the exercise will progress from bi-lateral to unilateral leg press. The training is performed with both the non-affected and the affected leg, although focus is on the affected leg. Repetition will be performed in three sets of 10 repetitions with an intensity of 12 repetition max. The participants are encouraged to perform the maximum number of repetitions possible within each series. If the number of repetitions are below 8 or exceeded 12, the loading will be adjusted for the next series.

Lying Leg Curl

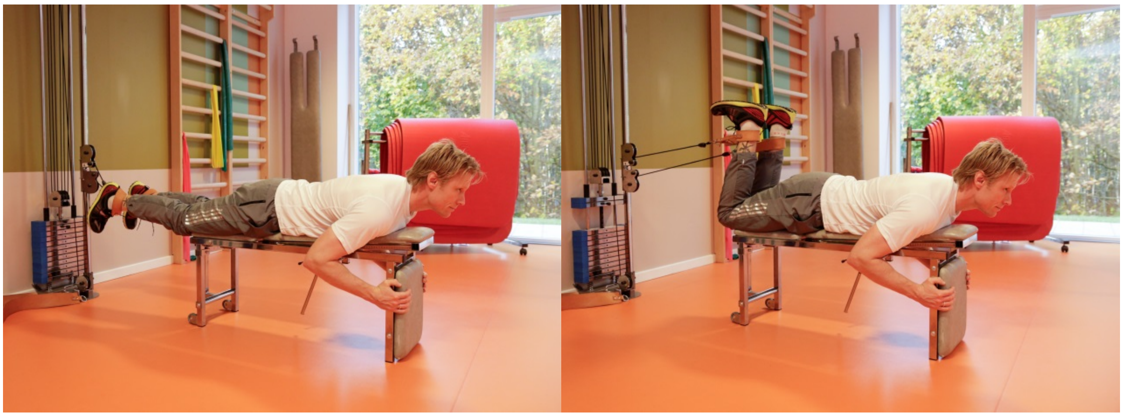

The machine lever is adjusted to fit the patient’s height; and the ankle straps are attached to both ankles from the low pulley attachments. The patient is instructed to lie prone on the bench with their knees just beyond the edge and to grip under the side or base of the bench for support. Their torso is kept flat on the bench to reduce hyperextension of their lower back, and to ensure their legs are fully extended. The patient’s toes are positioned straight. The patient is instructed to flex their legs as much as possible without lifting their thighs from the pad; and then to bring their legs back to the initial position. Focus will be on a fast/forceful knee flexion, followed by a slower and more controlled extending movement, towards starting position. When the patient can demonstrate full control in terms of performance, the exercise will progress from a bi-lateral to a unilateral leg press. The training is performed with both the non-affected and the affected leg, although focus is on the affected leg. Repetition will be performed in three sets of 10 repetitions with an intensity of 12 repetition max. The participants are encouraged to perform the maximum number of repetitions possible within each series. If the number of repetitions are below 8 or exceeded 12, the loading will be adjusted for the next series.

Squat

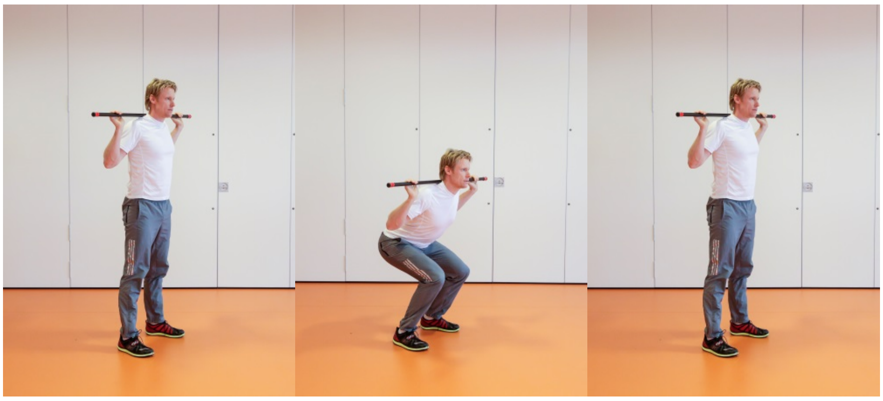

In the standing position, the patient begins with the barbell supported behind their head and on top of their shoulders. Their chest should be up and their head facing forward, their feet a shoulder width apart and turned out as needed. The patient is instructed to descend by flexing the knees, ensuring their knees and feet are aligned, keeping the weight on the front of the heel, and the torso as upright as possible. The patient is instructed to stop when their thighs are parallel with the floor, pause momentarily, then using their thighs and calves, return to the starting position. Focus will be on a fast/forceful knee extension, followed by a slower and more lean flexion movement towards starting position. Repetition will be performed in three sets of 10 repetitions with an intensity of 12 repetition max. The participants are encouraged to perform the maximum number of repetitions possible within each series. If the number of repetitions are below 8 or exceeded 12, the loading will be adjusted for the next series.

Nordic hamstring

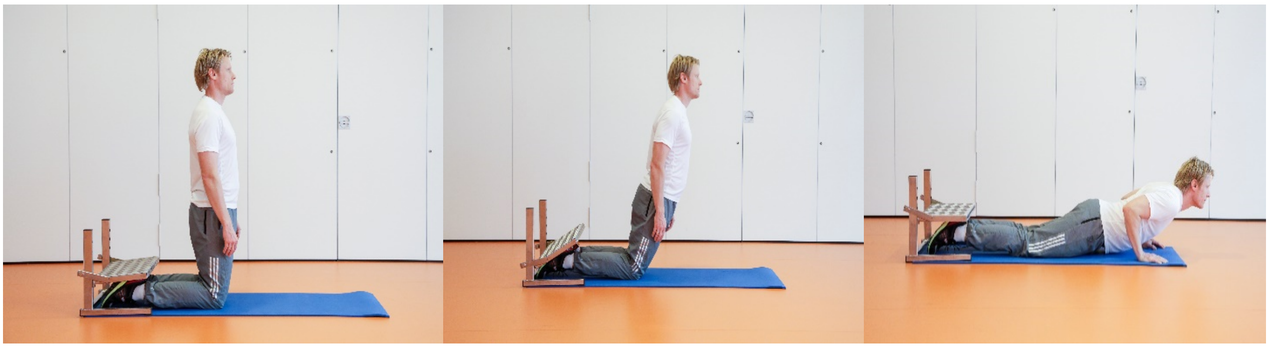

The patient starts in the kneeling position, with a straight upright torso, while accompanied by the instructor who helps stabilize the patient's lower legs on the mat. In an even movement, the patient leans forward as slowly as possible while resisting falling forward, keeping their hips and back in a linear position, reducing upper body acceleration with their arms to avoid falling; then pushing back with both arms to the starting position and repeating the exercise. After 1-2 weeks of familiarization (using an elastic band to reduce fast forward falling), progression will be completed in accordance with the protocol (see below).

Training protocol for Nordic Hamstring

WEEK	SESSIONS PER WEEK	SETS AND REPS.
1	1	2 x 5
2	1	2 x 8
3	2	2 x 6 - 8
4	2	3 x 6 - 8
5	2	3 x 8 - 10
6 - 10	2	3 sets, 12 – 10 - 8
11 - 12	1	3 sets, 12 – 10 - 8

Exercises with focus primarily on progressive neuromuscular training

Lunge progression

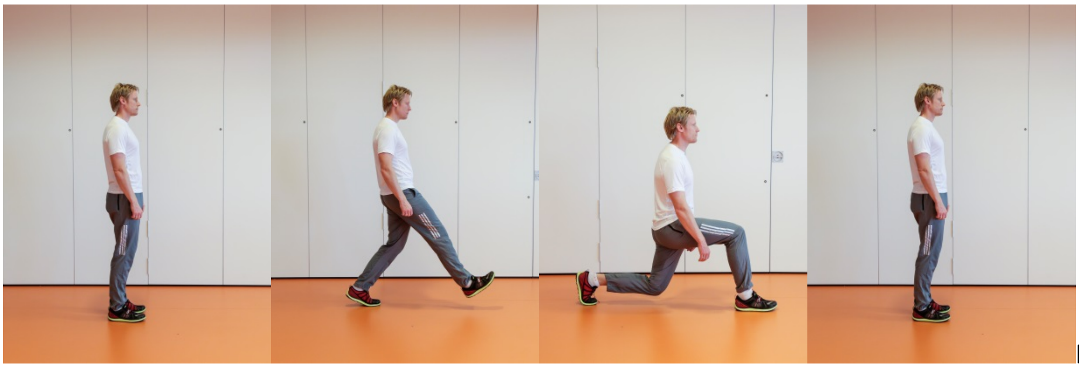
Phase 1

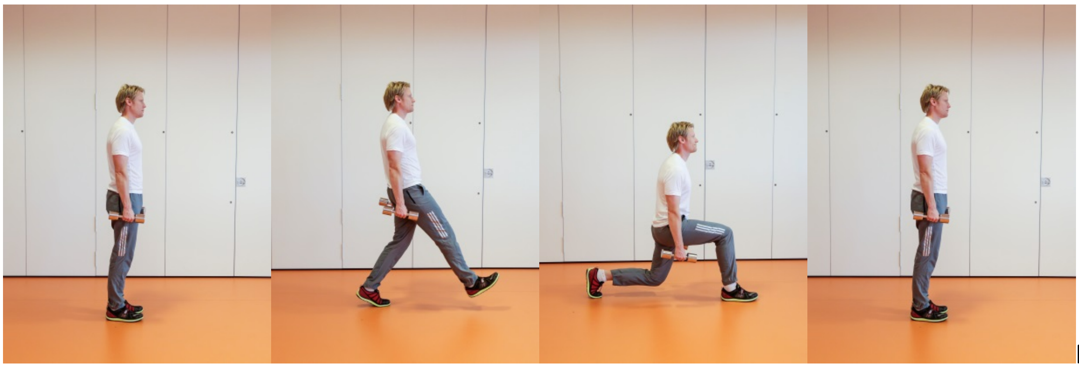
Phase 2

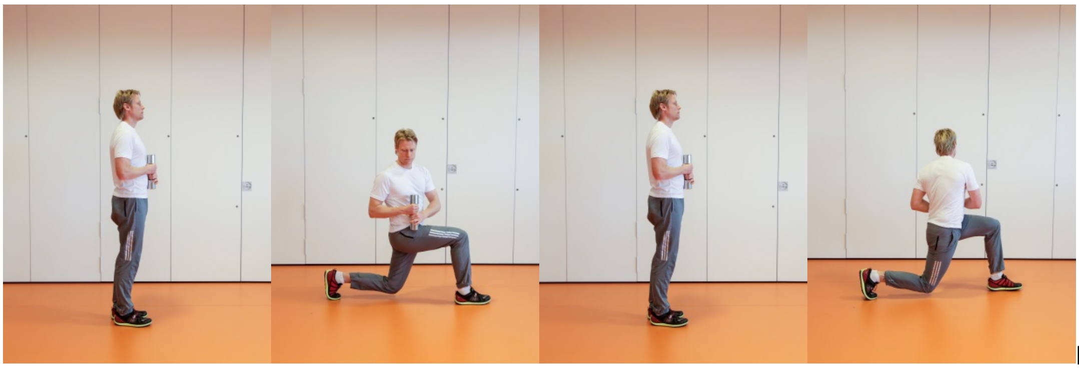
Phase 3

In a standing position, arms alongside the body and keeping the back straight throughout the exercise, the patient is instructed to step forward with the leg and lower the body until the knee of the rear leg is nearly in contact with the floor, maintaining most of the weight on the lead leg as the lunge progresses forward

into a deep knee flexion, while avoiding hyperextension of the trunk. The patient is instructed to return to the starting position and lunge forward with the opposite leg, then continue the lunges with alternating legs. Progress is made when a given exercise is performed with good sensorimotor control and a high quality of performance (based upon visual inspection by the physiotherapist). Phase 2 will maintain same focus, as mentioned above, although weights, in straight bearing, will be applied. Phase 3 will consist of all focus areas mentioned above. Furthermore, side-to-side upper body rotation will be applied for further neuromuscular severity. Number of sets, reps and weight (phase 2 – 3) will be recorded to determinate whether the patient is ready to progress, after each session. (Sets/reps before progression: Phase 1: 3 × 10 repetitions bilaterally; phases 2-3: 10 m × 2 sets bilaterally).

Lateral jump progression

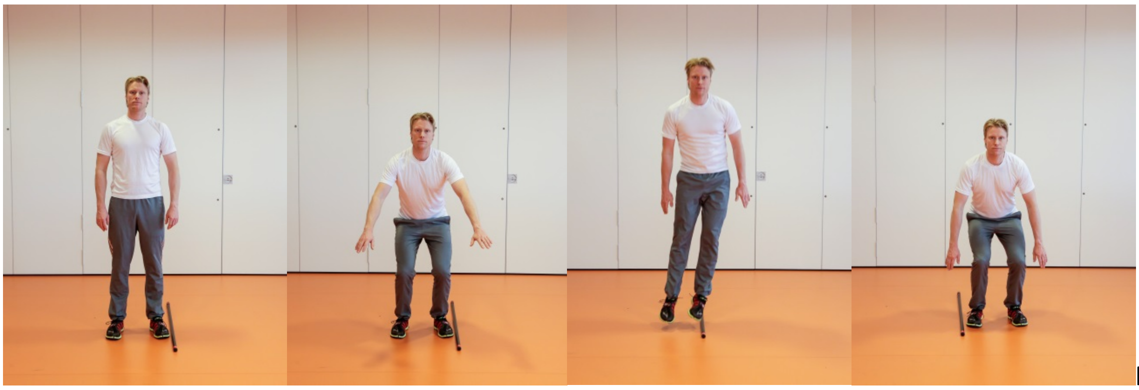

Phase 1

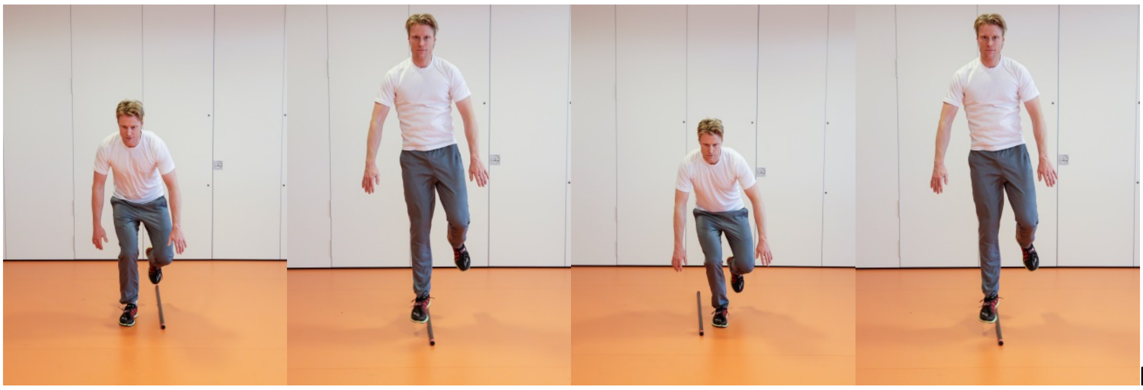

Phase 2 - 3

The patient stands straight, with elbows in the frontal plane, in line with the body. The patient is encouraged to jump “close to the line” in preparation for quicker lateral movements. The height of the jump is not the focus; rather, the criterion is good technique (i.e. alignment of the hip, knee and ankle in the frontal plane), increasing speed and power in take-off from the floor. A deep knee flexion position is emphasized upon each take-off and landing, regardless of the phase. This exercise progresses from double leg (phase 1) to single leg (phase 2 and 3), when the patient can demonstrate symmetrical timing and proper alignment in all sets/reps in the current phase (phases 1: 2 × 10 repetitions; phase 2: 2 × 10 repetitions (for each leg); phase 3: 3 × 10 repetitions (for each leg).

Prone trunk stability

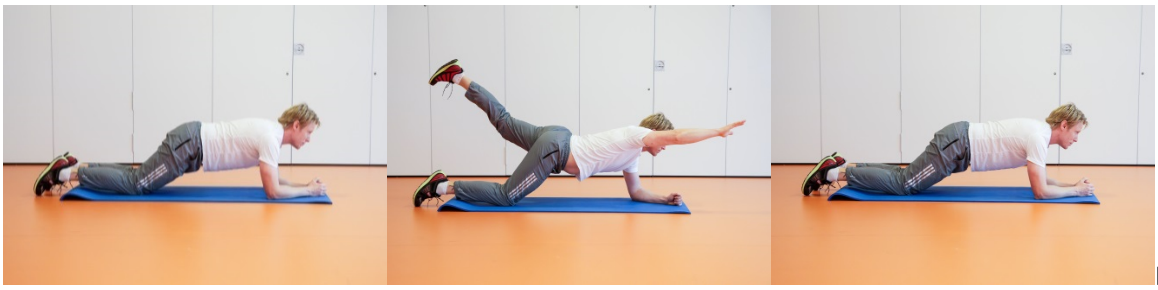

Phase 1

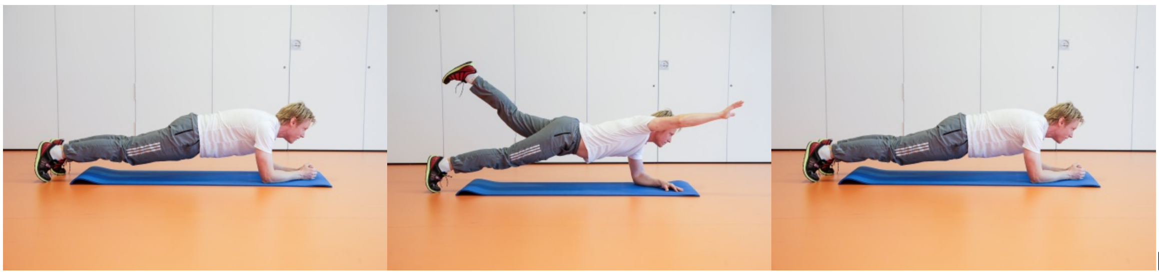
Phase 2

In the prone position, the patient lifts their torso, supporting their body weight on their forearms and lower legs, while maintaining their back in a straight position. The patient is instructed to raise their opposite leg/arm in a linear movement. As the patient progresses to the prone bridge position (phase 2), the contact points away from the center of mass further destabilize the patient as they alternate extremity limb positions. The goal is to avoid excessive trunk rotation and flexion or hyperextension when the limbs are lifted. This exercise progresses, when the patient can demonstrate symmetrical timing and proper alignment in all sets/reps in the current phase (phase 1 - 2: 3 × 10 repetitions bilaterally).

Posterior chain progression

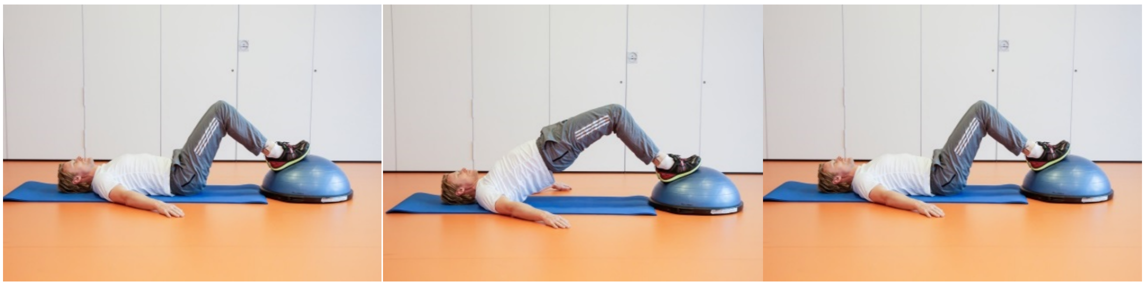
Phase 1

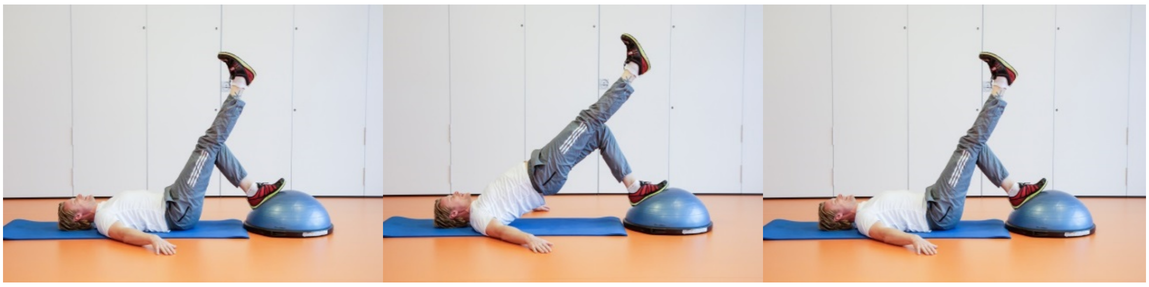

Phase 2

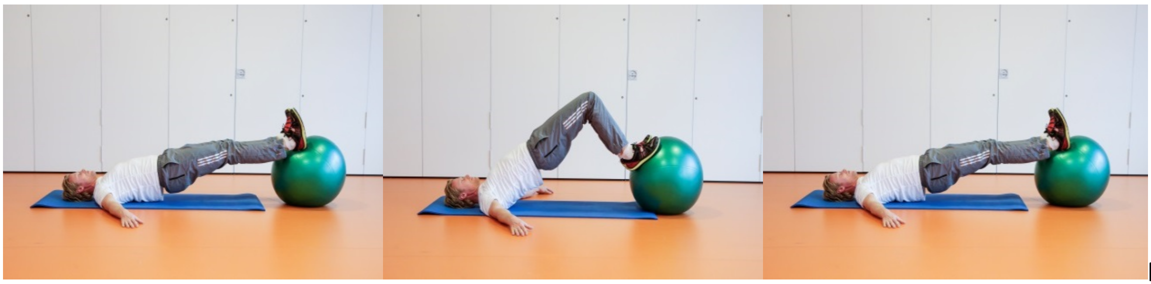

Phase 3

The patient lies supine on the floor with their lower legs on an exercise ball and their arms extended out to the side. The patient is instructed to bend their knees and hips, and to raise their lower back and hips off the floor, avoiding lumbar hyperextension during the bridging task phases. As the patient advances through the phases, the goal is to perform full, uncompensated motion.

In phase 3, the patient is instructed to keep their hips straight and knees bent, while their heels are pulled backwards, allowing their feet to roll onto the ball. The patient is then instructed to lower their body to the original position by straightening their knees, while minimizing the motion of the ball under their feet and achieving controlled hip flexion and extension. This exercise progress, when the patient can demonstrate symmetrical timing and proper alignment in all sets/reps in the current phase (All phases: 3 × 10 repetitions).
