# Supplementary material for: The effect of targeted exercise on knee-muscle function in patients with persistent hamstring deficiency following ACL reconstruction – study protocol for a randomized controlled trial
Source: Trials. 2018 Jan 26;19:75. doi: 10.1186/s13063-018-2448-3 (PMC5787267; doi:10.1186/s13063-018-2448-3)
Supplement: Supplementary file 3 — Exercise protocol. Exercise protocol for home-based intervention group. (PDF 286 kb) [file 13063_2018_2448_MOESM3_ESM.pdf]

Appendix 2.

Control Group

Gluteal press

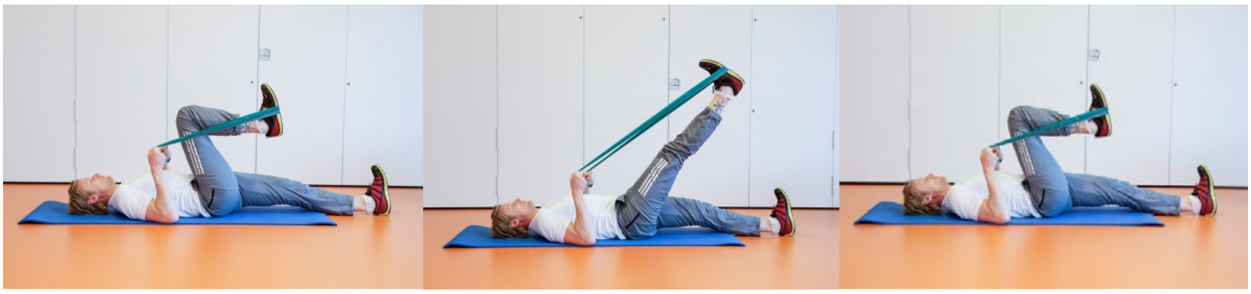

The patient lies on their back with both legs extended and arms bent by their sides, while holding the ends of an elastic band, which is placed under the arch of one of their feet. The patient is instructed to pull on the elastic band, draw their knee to their chest, press their elbows into the ground and lift their hips off the floor, as their leg extends out straight at a 45-degree angle. The hips are then lowered to the floor and the knee is pulled to the chest ready for the next repetition. (10 unilateral repetitions x 3 for each leg).

Squats

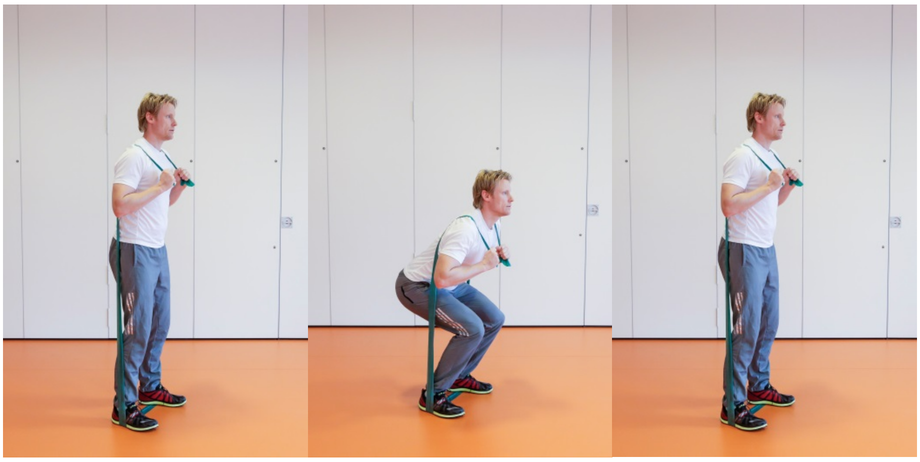

The patient stands on the elastic band with both feet, hips width apart. The ends of the elastic band are held in each hand, then drawn up and over the back of the shoulders then held tightly at chest height. The back, chest and head are kept straight and the elbows back. The patient is instructed to bend their legs and squat down until their thighs are parallel with the floor, to pause momentarily, and then using their thighs and calves, to return to the starting position. (10 repetitions with the elastic band as tight as possible).

Leg curl (standing)

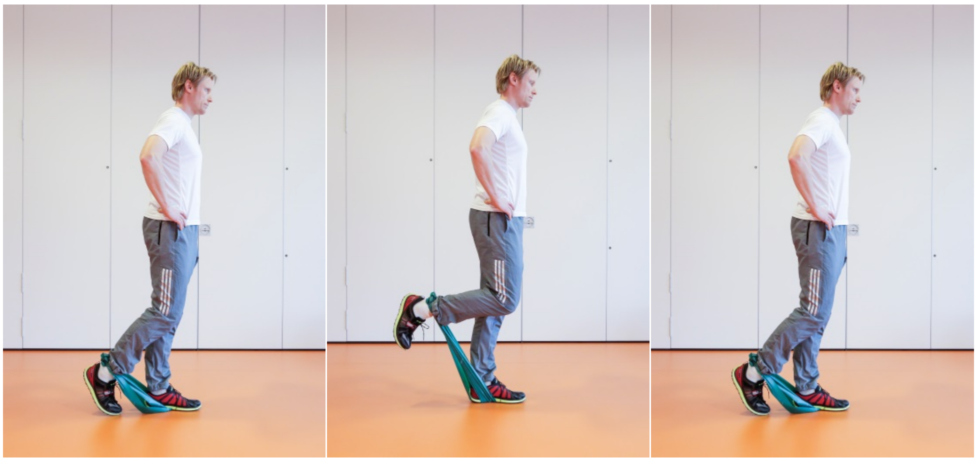

The elastic band is doubled then wrapped around one ankle and the patient stands on the ends of the elastic band with the other foot. The body is kept upright and the hands are placed on the hips (or used to keep balance). The patient is instructed to flex the free leg until the lower leg is parallel to the floor or slightly higher (approx. 90 degrees). The elastic band is folded to keep it as tight as possible. (10 unilateral repetitions x 3 for each leg).

Band stationary lunge

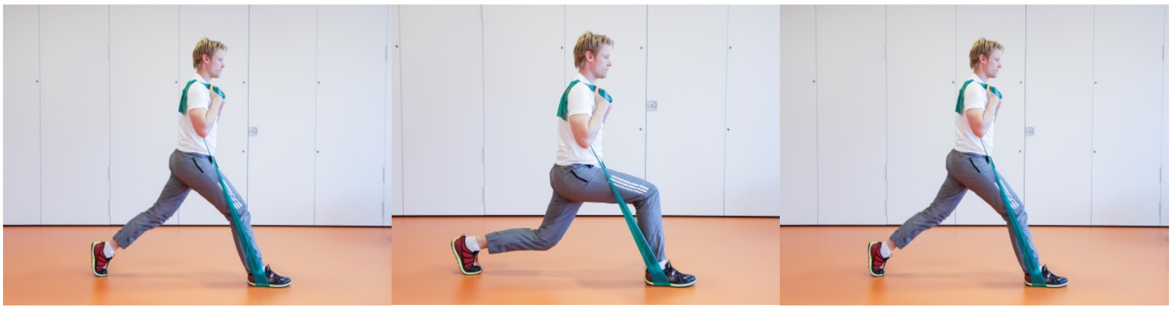

The elastic band is placed under the arch of one foot. The ends of the elastic band are held in each hand, then drawn up and over the back of the shoulders then held tightly at chest height. The back, chest and head are kept straight and the elbows back. The patient lunges forward with the leg that has the elastic band and lowers the body until the knee of the rear leg is nearly in contact with the floor, maintaining most of the weight on the lead leg as the lunge progresses forward into a deep knee flexion and avoiding hyperextension of the trunk. The patient is instructed to return to the starting position and lunge forward with the opposite leg, and then continue the lunges with alternating legs. (10 unilateral repetitions x 3 for each leg).
